# Augmentierte Primärnaht „internal bracing“ nach ligamentärer Ellenbogenluxation

**DOI:** 10.1007/s00064-022-00788-1

**Published:** 2022-12-05

**Authors:** Valentin Rausch, Matthias Königshausen, Thomas A. Schildhauer, Jan Geßmann

**Affiliations:** Chirurgische Universitätsklinik und Poliklinik, BG Universitätsklinikum Bergmannsheil Bochum, Ruhr-Universität Bochum, Bürkle-de-la-Camp Platz 1, 44789 Bochum, Deutschland

**Keywords:** Ellenbogen, Ellenbogenluxation, Internal bracing, Primärnaht, Luxation, Elbow, Elbow dislocation, Internal bracing, Primary suture, Dislocation

## Abstract

**Operationsziel:**

Ziel der operativen Versorgung ist die primäre Stabilisierung des instabilen Ellenbogens nach ligamentärer Ellenbogenluxation.

**Indikationen:**

Ligamentäre Ellenbogenluxationen werden von unterschiedlichen Verletzungen der umgebenden Muskulatur sowie der Kollateralbänder begleitet. Die operative Versorgung ist bei Versagen der konservativen Therapie indiziert, d. h. wenn eine Dezentrierung oder Reluxation nur durch Ruhigstellung in > 90° Beugung und Pronation verhindert werden kann oder das Gelenk durch aktive muskuläre Führung nach 5 bis 7 Tagen nicht zentriert werden kann.

**Kontraindikationen:**

Kontraindikationen für eine alleinige augmentierte Primärnaht bestehen in der Regel bei begleitenden knöchernen Verletzungen im Rahmen der Luxation, bei ausgedehnten Weichteilverletzungen sowie im Falle eines Infektes am Ellenbogen.

**Operationstechnik:**

Die Versorgung mittels augmentierter Primärnaht des Ellenbogens erfolgt kombiniert über einen lateralen (Kocher) und medialen (FCU-Split) Zugang zum Ellenbogen. Nach Reposition des Ellenbogens werden jeweils zuerst die Kollateralbänder mit hochfesten Polyethylenfäden augmentiert und gemeinsam mit einem weiteren hochfesten Polyethylenfaden im Humerus verankert. Darüber werden die Extensoren bzw. Flexoren ebenfalls fadenaugmentiert am Epicondylus lateralis bzw. medialis refixiert.

**Weiterbehandlung:**

Ziel der Weiterbehandlung ist die frühfunktionelle Beübung des Ellenbogens, die in einer Bewegungsorthese unter Vermeidung von Varus- und Valgusbelastung erfolgt.

**Ergebnisse:**

Im Zeitraum zwischen August 2018 und Januar 2020 wurden insgesamt 12 Patienten mit einer augmentieren Primärnaht nach instabiler rein ligamentärer Ellenbogenluxation versorgt. Nach einem mittleren Nachverfolgungszeitraum von 14 ± 12,7 Monaten zeigte sich ein Mayo-Elbow Performance Score von im Mittel 98,5 Punkten bei mittlerem funktionellem Bogen von 115°. Keiner der Patienten gab ein verbleibendes Instabilitätsgefühl des Ellenbogens an.

## Abstract (en)

**Objective:**

Aim of surgical treatment is the primary stabilization of the unstable elbow following a ligamentous elbow dislocation.

**Indications:**

Ligamentous elbow dislocations are typically accompanied by injuries to the surrounding musculature and collateral ligaments of the elbow joint. Surgical treatment is indicated in case of failure of nonoperative therapy, i.e., when a dislocation can only be prevented in immobilization > 90° and pronation of the elbow or an active muscular centering of the elbow fails after 5–7 days.

**Contraindications:**

Contraindications for a solely “internal bracing” augmented primary suture are generally in the case of accompanying bony injuries in elbow dislocations, extensive soft-tissue injuries, and septic arthritis of the elbow.

**Surgical technique:**

The augmented primary suture of the elbow is performed using both a lateral (Kocher or Kaplan) and medial (FCU split) approach to the elbow. After reduction of the elbow, the collateral ligaments are first augmented with high-strength polyethylene suture and fixed in the distal humerus together with another high-strength polyethylene augmentation suture. The extensors and flexors are then fixed to the medial and lateral epicondyle, respectively, using suture anchors.

**Postoperative management:**

The aim of the postoperative management is early functional exercise of the elbow. The elbow is placed in an elbow brace to avoid varus and valgus load.

**Results:**

Between August 2018 and January 2020, a total of 12 patients were treated with an augmented primary suture following unstable ligamentous elbow dislocation. After a mean follow-up of 14 ± 12.7 months, the mean Mayo Elbow Performance Score was 98.5 points with a mean functional arc of 115°. None of the patients reported a recurrent dislocation or persistent instability of the elbow.

## Vorbemerkungen

Luxationen des Ellenbogens treten mit einer Inzidenz von ca. 5/100.000 verhältnismäßig häufig auf [25]. Insgesamt sind Frauen ähnlich häufig wie Männer betroffen, in den ersten Lebensdekaden treten Ellenbogenluxationen allerdings häufiger bei Männern auf, während sich das Verhältnis ab der 4. Lebensdekade umkehrt [25]. Hierfür sind häufig Sportunfälle oder Stürze auf den ausgestreckten Arm oder seltener direkte Anpralltraumata verantwortlich [25]. Gängige Theorien zur Abfolge der Verletzungen bei einer Ellenbogenluxation sind widersprüchlich, gehen aber am ehesten von einer sequenziellen Verletzung der stabilisierenden Strukturen aus. Die populärste Theorie stammt von O’Driscoll und propagiert einen lateralen Beginn der Verletzung durch eine Kombination aus axialer Stauchung, Valgus- und Supinationskraft [19]. Bildgebende Untersuchungen konnten diese Abfolge aber nicht durchgehend rekonstruieren, sodass der primäre Verletzungsfokus wahrscheinlich am ehesten von der individuell einwirkenden Kraft abhängig ist und nicht verallgemeinert werden sollte [16, 21, 22, 24]. Die Klassifikation der Ellenbogenluxation richtet sich (a) nach der knöchernen Beteiligung und (b) der Richtung der Luxation, die allerdings in der weit überwiegenden Zahl der Fälle nach posterior geht. Liegen keine knöchernen Begleitverletzungen vor, wird die Luxation als rein ligamentäre Luxation, andernfalls als knöcherne Ellenbogenluxation klassifiziert. Synonym werden auch die Begriffe der „einfachen“ Luxation für rein ligamentäre oder „komplexe“ bei knöcherner Beteiligung verwendet [12]. Diese Bezeichnungen können allerdings über den Schweregrad ligamentärer Ellenbogenluxationen hinwegtäuschen und werden daher in der eigenen Praxis nicht verwendet. Ligamentäre Begleitverletzungen können von isolierten Verletzungen der Kapsel, des lateralen oder medialen Kollateralbandes hin zu Kombinationsverletzungen mit vollständigem Abriss der gemeinsamen Muskelinsertionen am medialen oder lateralen Epikondylus sowie der Ellenbogen-überschreitenden Muskulatur wie beispielsweise dem M. brachialis reichen [1].

Generell ist die rein ligamentäre Ellenbogenluxation eine Domäne der konservativen Therapie. Nach unverzüglicher Reposition (ggf. unter Analgosedierung) kann eine kurze Ruhigstellung mit folgender frühfunktioneller Therapie zu guten klinischen Ergebnissen mit niedrigen Raten sekundärer operativer Eingriffe führen [2, 10, 17, 18]. Die Prognose der Luxation hängt allerdings auch von dem Verletzungsausmaß der umgebenden Weichteilstrukturen ab, wobei allerdings unklar ist, welche Patienten von einer primären operativen Versorgung profitieren und mit welchem verbleibenden Ausmaß einer Instabilität gerechnet werden muss [1]. Eine konservative Therapie bedingt außerdem eine aktive muskuläre Stabilisierung, die bei schwereren Verletzungen kompromittiert sein kann.

Für die operative Versorgung stehen verschiedene Techniken zur Verfügung, bei denen in der akuten Phase in der Regel eine Refixierung des verletzten Kollateralbandapparates und der Extensoren und Flexoren im Vordergrund steht, die bei verbleibender Instabilität mit einem Fixateur externe kombiniert werden kann [7, 14]. Die Verwendung eines Fixateur externe bei instabilem Ellenbogen hat prinzipiell den Vorteil der schnellen und einfachen Anwendbarkeit, insbesondere in der Akutversorgung. Zudem kann ein Fixateur auch als Bewegungsfixateur angelegt werden und somit die Beweglichkeit des Ellenbogens erhalten bleiben. Allerdings ist die Anlage eines Bewegungsfixateurs technisch in Anlage und Nachbehandlung ungleich anspruchsvoller als ein Fixateur ohne Bewegungsmodul, ist sehr unkomfortabel für den Patienten und erfordert einen zweizeitigen Abbau des Fixateurs. Klinische Daten für die Verwendung eines Bewegungsfixateurs sind nur unzureichend vorhanden und erlauben daher keine allgemeine Empfehlung [13].

Die alleinige Refixierung der Kollateralbänder ähnelt der des „internal bracing“ mit separatem medialem und lateralem Zugang. Die Versorgung mit additiven, nichtresorbierbaren hochfesten Polyethylenfäden bietet allerdings ähnlich der Versorgung nach Instabilitäten an anderen Gelenken die Möglichkeit der Augmentation der Refixation, um eine biomechanisch stabilere Ausheilung zu gewährleisten [11, 15]. Biomechanische Arbeiten zum „internal bracing“ am Ellenbogen zeigen im Vergleich zu Rekonstruktionstechniken mit Sehnentransplantation eine vergleichbare Stabilität am medialen und lateralen Ellenbogen, die einer isolierten Refixierung ohne Augmentation biomechanisch signifikant überlegen sind. Bei frühfunktioneller Nachbehandlung können diese Techniken aber auch bei höhergradiger Instabilität zu guten klinischen Ergebnissen führen [3, 5, 8, 9, 20].

## Operationsprinzip und -ziel

**Figure 1:**
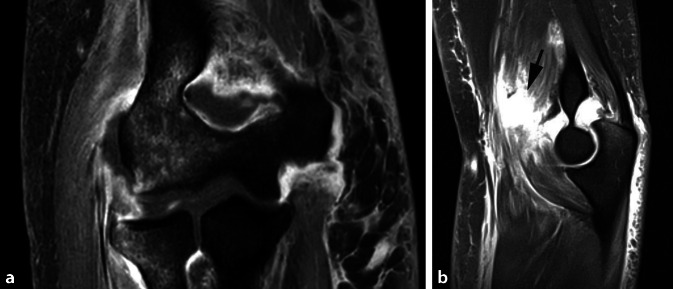

**Figure 2:**
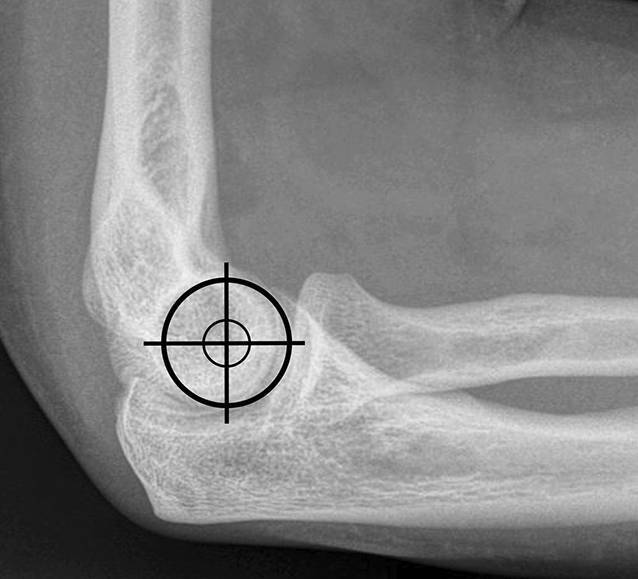

Bei hochgradiger anhaltender Instabilität des Ellenbogens mit Reluxation des Gelenks oder Subluxation bei bestehender Ruptur der gemeinsamen Extensoren- und Flexorenansätze des Ellenbogens eignet sich eine augmentierte Primärnaht, um eine stabile Gelenkführung für eine Ausheilung der verletzten Strukturen zu gewährleisten (Abb. 1). Ziel der augmentierten Primärnaht ist daher die anatomische Rekonstruktion der ausgerissenen Kollateralbänder im näherungsweisen Rotationszentrum des Ellenbogens sowie der Extensoren- und Flexorenansätze und eine kongruente Gelenkstellung über den funktionellen Bogen (Abb. 2). Kann eine stabile Gelenkführung gewährleistet werden, sollte frühzeitig eine funktionelle Nachbehandlung begonnen werden, um eine mögliche Ellenbogengelenksteife zu verhindern. Wir empfehlen die augmentierte Primärnaht bei Ellenbogenluxationen bei (a) Dezentrierung bzw. Subluxation nach Reposition in der Gipsschiene oder Halten der reponierten Stellung nur in Pronation und Beugung über 90°, (b) klinischer Unfähigkeit der aktiven muskulären Führung unter Aufhebung von Varus- und Valgusstress möglichst über das volle Bewegungsausmaß nach Luxationsereignis oder anhaltender Dezentrierung bzw. Subluxation in den Verlaufsröntgenbildern. Bildmorphologische Befunde der stabilisierenden Strukturen werden nicht isoliert für die operative Versorgung verwendet. Die beschriebene Operationstechnik des „internal bracing“ am Ellenbogen orientiert sich an der publizierten Technik von Bhide et al. [4].

## Vorteile

- Die augmentierte Primärnaht der Kollateralbänder, Extensoren und Flexoren am Ellenbogen erlaubt nach Ellenbogenluxation auch bei hochgradiger Instabilität die frühzeitige Wiederherstellung der Stabilität und damit eine frühfunktionelle Behandlung.
- Der Vorteil gegenüber einer alleinigen Primärnaht der Kollateralbänder besteht in der überlegenen biomechanischen sofortigen Stabilität, die einer Rekonstruktion mit einem autologen oder allogenen Sehnentransplantat biomechanisch nicht unterlegen ist.
- Gegenüber der Behandlung mit einem Fixateur externe bietet die augmentierte Primärnaht v. a. einen deutlich höheren Patientenkomfort mit der Möglichkeit der frühfunktionellen Behandlung.
- Eine bestehende Gelenkinkongruenz als Zeichen der Instabilität kann damit primär aufgehoben werden.

## Nachteile

- Mögliche Irritationen durch einliegendes Fadenmaterial
- Technisch anspruchsvoll, da die Möglichkeit der Fehlplatzierung der Anker mit potenzieller Dezentrierung des Gelenks besteht
- Allgemeine Operationsrisiken

## Indikation

- Ellenbogenluxation mit unmittelbarer Reluxation auch unter Ruhigstellung im Oberarmgips > 90° und Pronation oder anhaltender Dezentrierung im Oberarmgips, wobei die Richtung der Luxation nicht für die Indikation entscheidend ist
- Fehlende Möglichkeit der aktiven muskulären Zentrierung des Ellenbogens nach 5 bis 7 Tagen
- Verbleibende Instabilität des Ellenbogens nach Stabilisierung knöcherner Strukturen bei knöchern ligamentären Luxationen

## Kontraindikation

- Inoperabilität aufgrund von Begleitverletzungen/-erkrankungen
- Eine alleinige augmentierte Primärnaht ist bei knöchernen Begleitverletzungen des Ellenbogens in der Regel nur bei kleineren (undislozierten) knöchernen Absprengungen beispielsweise des Processus coronoideus oder des Radiuskopfes indiziert
- Ausgeprägte Arthrose des Ellenbogengelenks
- Ausgedehnte Weichteilverletzungen
- Ellenbogengelenkinfektion

## Patientenaufklärung

- Allgemeine Operationsrisiken
- Ellenbogengelenksteife mit möglicher folgender Arthrolyse
- Möglichkeit des Entstehens heterotoper Ossifikationen mit folgender Bewegungseinschränkung
- Verbleibende Instabilität
- Verletzung des N. ulnaris
- Posttraumatische Arthrose

## Operationsvorbereitung

- Konventionelle Röntgenaufnahmen des Ellenbogens (a.-p. und lateral) vor und nach Reposition des Ellenbogens
- Wir verwenden die MRT-Diagnostik regelhaft bei jeder Ellenbogenluxation, insbesondere um das Verletzungsausmaß des medialen und lateralen Kollateralbandkomplexes sowie der stabilisierenden Muskulatur am Ellenbogen (insbesondere Extensoren und Flexoreninsertionen am lateralen bzw. medialen Epikondylus sowie M. brachialis, M. biceps brachii und M. triceps brachii) zu quantifizieren. Bei fehlender stabiler Führung oder Zeichen der Gelenkinkongruenz des Ellenbogens (beispielsweise „drop-sign“) kann die MRT zur Beurteilung der stabilisierenden Strukturen verwendet werden. Allerdings wird die Indikation zur Operation nicht allein an morphologischen Veränderungen in der MRT gestellt. Bei der MRT-Diagnostik der Bandstrukturen wird generell eine ausgestreckte Stellung des Armes über dem Kopf präferiert, da hierbei die Bandstrukturen besser beurteilt werden können. Dies ist aber in der Regel schmerz- und stabilitätsbedingt nicht möglich, eine gröbere Verletzung an den Strukturen ist in der Regel aber auch in Beugestellung möglich. Bei knöchernen Verletzungen führen wir ergänzend eine CT des Ellenbogens durch.
- Neurologischer Status vor und nach Reposition sowie Untersuchung der peripheren Motorik und Sensibilität
- Untersuchung des Ellenbogengelenks in Bezug auf Voroperationen und Weichteile des Ellenbogens. Bei ausgedehnten Weichteildefekten oder (seltenen) offenen Luxationen sollte primär ein Fixateur externe angelegt werden.
- Single-shot-Antibiose (beispielsweise Cefazolin 2 g i.v. nach Ausschluss von Allergien) in der Einleitung

## Instrumentarium

**Figure 3:**
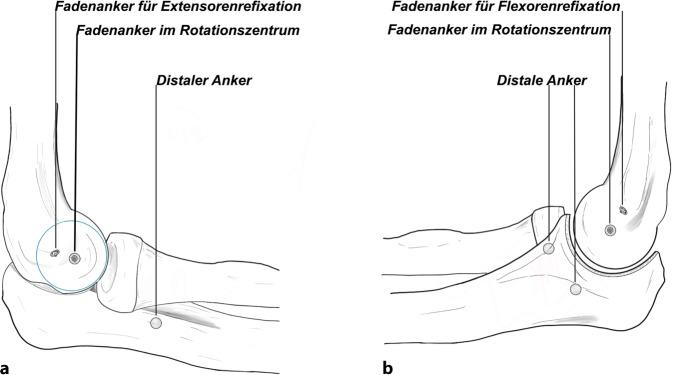

- Standard-Knocheninstrumentarium
- Verschiedene Fadenanker zur Fixation der augmentierten Primärnaht: (1) 3,5 mm Interferenzschraube zur humeralen Fixation (beispielsweise BioComposite SwiveLock-Anker, Fa. Arthrex, München, Deutschland), (2) Polyethylen-tape-beladene All-suture-Anker für den Augmentationsfaden ulnar (beispielsweise ICONIX, Fa. Stryker, Portage, MI, USA), (3) Titan-Fadenanker zur Refixation der gemeinsamen Extensoren- bzw. Flexorenansätze an den Epikondylen (beispielsweise GII-Anker, Fa. Mitek, Raynham, MA, USA; Abb. 3).

## Anästhesie und Lagerung

**Figure 4:**
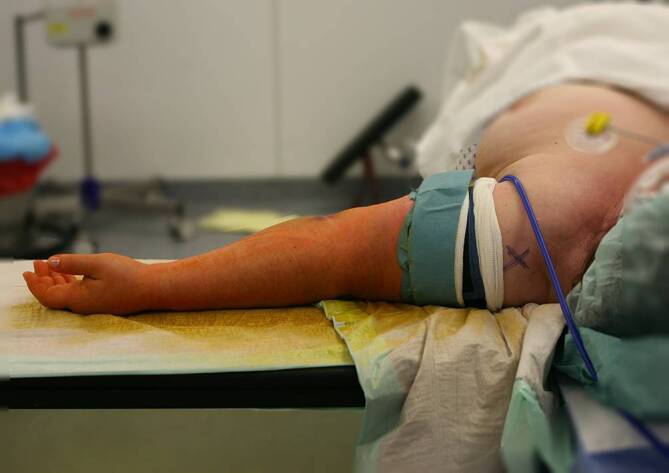

- Allgemeinanästhesie, möglichst in Kombination mit Regionalanästhesie
- Der Patient wird in Rückenlage gelagert und der Arm auf einem Armtisch ausgelagert (Abb. 4)
- Anlegen der Blutsperre, steriles Abdecken (beispielsweise mit einem Lochtuch)

## Operationstechnik

**Figure 5:**
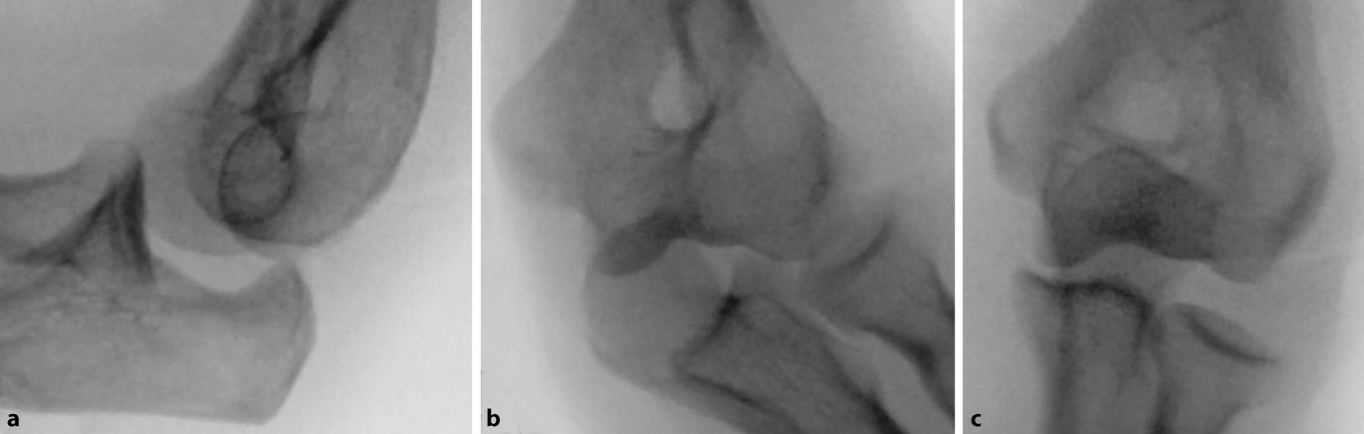

**Figure 6:**
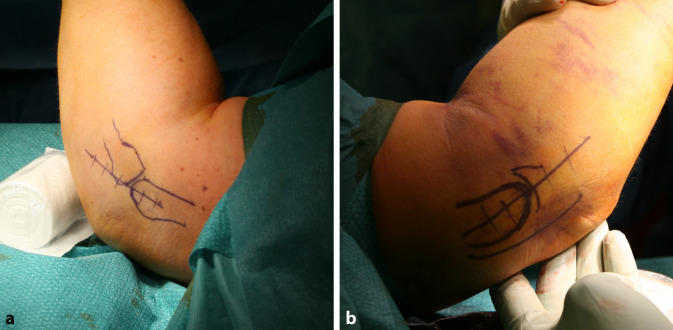

**Figure 7:**
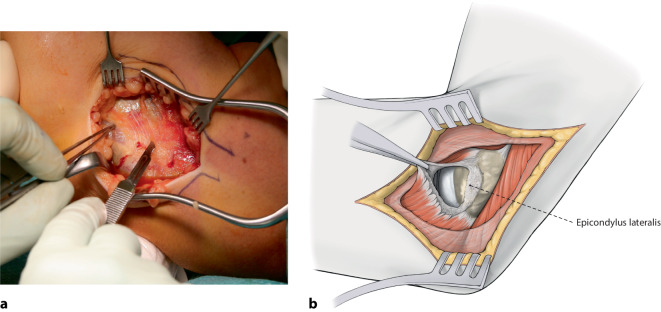

**Figure 8:**
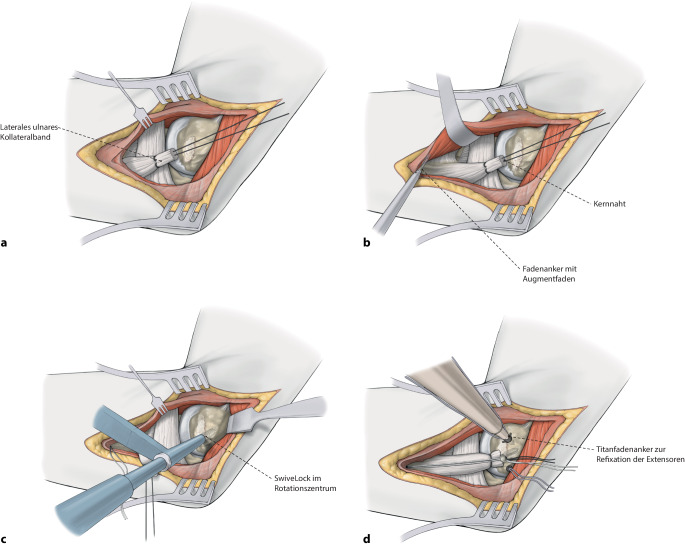

**Figure 9:**
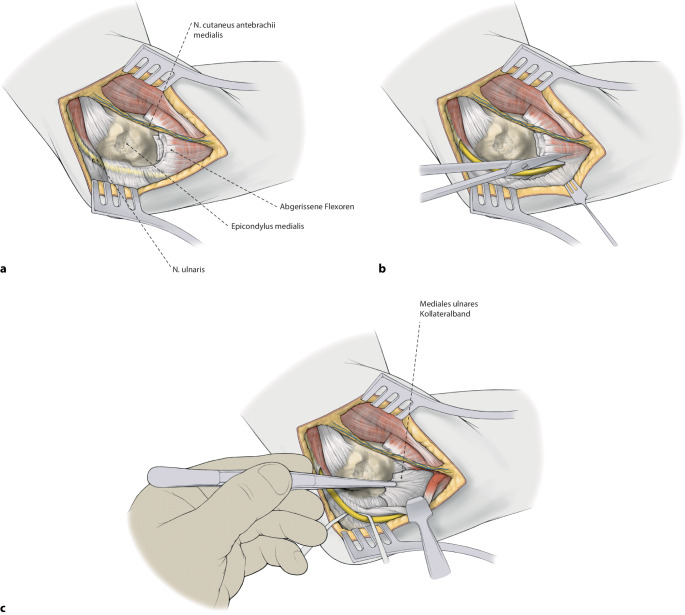

**Figure 10:**
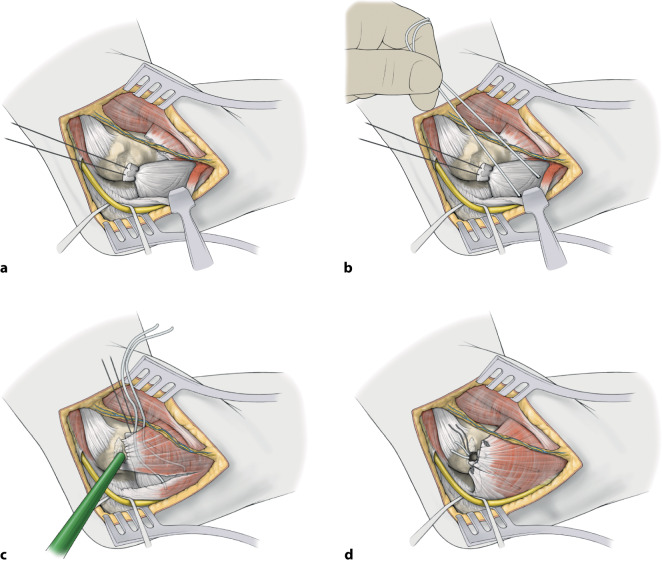

**Figure 11:**
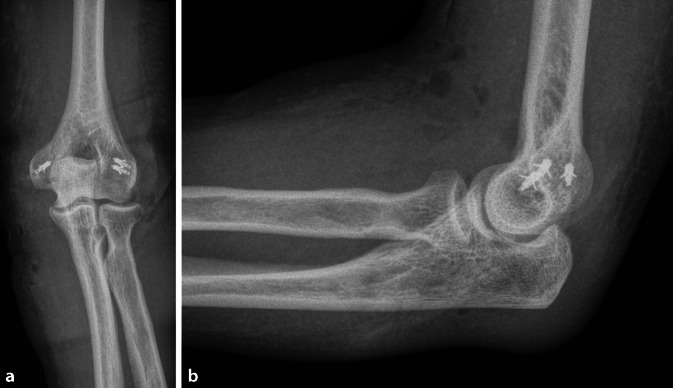

(Abb. 5, 6, 7, 8, 9, 10, 11)

## Postoperative Behandlung

- Intraoperativ dynamische Kontrolle der Stabilität unter dem Bildwandler, postoperativ radiologische Stellungskontrolle im a.-p.- und lateralen Strahlengang
- Bedarfsgerechte postoperative Analgesie nach WHO-Stufenschema
- Bei stabiler Führung in der Bildwandlerkontrolle frühfunktionelle Therapie ohne Belastung des Ellenbogens in Bewegungsorthese unter Vermeidung von Varus- und Valgusbelastung. Extension und Flexion werden in der Bewegungsorthese direkt freigegeben. Das Handteil der Orthese wird ebenfalls entfernt, sodass auch Pro- und Supination freigegeben sind.
- In der Regel wird unmittelbar postoperativ eine aktiv-assistive Beübung im Overhead-Motion-Protokoll empfohlen [23]. Hierfür wird der Oberarm über den Kopf gebracht, die Flexion des Ellenbogens erfolgt mit der Schwerkraft. Bei guter Schmerzkontrolle kann der Ellenbogen auch direkt aktiv beübt werden.
- Belastungsaufbau ab 6 Wochen postoperativ und fehlender Beschwerdesymptomatik. Nach 6 Wochen wird der Ellenbogen zudem radiologisch verlaufskontrolliert und die Bewegungsorthese abgenommen.
- Eine medikamentöse Prophylaxe zur Verhinderung heterotoper Ossifikationen wird aufgrund fehlender Evidenz nicht generell empfohlen. Sollten Risikofaktoren für die Entwicklung heterotoper Ossifikationen vorliegen (Verletzungen des ZNS, größere Verbrennungen oder bekannte Vorerkrankungen wie Morbus Paget) kann aber eine medikamentöse Prophylaxe beispielsweise mit Indometacin oder Ibuprofen erwogen werden.

## Fehler, Gefahren und Komplikationen

- Rezidivinstabilität durch Versagen der primären Naht oder Dislokation der Fadenkanker: Revision der Naht möglichst mit Neuplatzierung und ggf. additivem Fixateur externe
- Sekundäre Ellenbogensteife durch Narbengewebe oder heterotope Ossifikationen: arthroskopische oder offene Arthrolyse (je nach Expertise und intra- bzw. extraartikulärer Ursache)
- Heterotope Ossifikationen: prophylaktische Behandlung beispielsweise mit Ibuprofen oder Indometacin, bei ausgeprägten heterotopen Ossifikationen offene Arthrolyse mit Resektion der Ossifikationen und ggf. Bestrahlungstherapie
- Verletzungen oder Irritationen des N. ulnaris: routinemäßige Darstellung und Schonung des N. ulnaris intraoperativ
- Irritationen durch eingebrachtes Fadenmaterial: je nach Schwere der Irritationen Entfernung von prominentem Fadenmaterial nach stabiler Einheilung
- Frühinfekt des Ellenbogengelenks nach operativer Versorgung: offene Spülung, Débridement und resistenzgerechte antibiotische Therapie
- Die besten Ergebnisse werden mit einer frühfunktionellen Therapie erzielt. Der Patient sollte über eine ausführliche funktionelle Beübung des Ellenbogens unter Vermeidung von Varus- und Valgusbelastung bei anliegender Orthese aufgeklärt werden. Der Patient sollte außerdem darüber aufgeklärt werden, dass der Ellenbogen für 6 Wochen nicht belastet werden darf.

## Ergebnisse

Im Zeitraum zwischen August 2018 und Januar 2020 wurden insgesamt 12 Patienten mit einer augmentieren Primärnaht nach instabiler ligamentärer Ellenbogenluxation versorgt (Tab. 1). In 10 Fällen wurde sowohl der mediale wie auch der laterale Kollateralbandapparat adressiert, in 2 weiteren Fällen isoliert der mediale Kollateralbandapparat, da nach medialer Stabilisierung keine höhergradige laterale Instabilität vorlag. In der Regel waren neben dem medialen und lateralen Kollateralbandapparat auch die Ansätze der Flexoren und Extensorenmuskulatur beteiligt. Zehn Patienten standen zur Nachuntersuchung zur Verfügung. Nach einem mittleren Nachverfolgungszeitraum von 14 ± 12,7 Monaten (Spannweite: 1 bis 34 Monate) zeigte sich ein Mayo-Elbow Performance Score von im Mittel 95,5 ± 2,3 Punkten bei einem mittleren funktionellen Bogen von 115 ± 24,5° (Spannweite: 70–150°). Keiner der Patienten gab ein verbleibendes Instabilitätsgefühl des Ellenbogens an. In einem Fall musste aufgrund einer sekundären Ellenbogensteife eine offene Arthrolyse durchgeführt werden.

**Table 1:** 

Alter	Geschlecht	Zugang	MEPS	Funktioneller Bogen (°)	Pro‑/Supination (°)
76	Weiblich	Kocher + FCU-Split	100	110	90-0-70
74	Weiblich	Kocher + FCU-Split	100	110	90-0-90
58	Weiblich	Kocher + FCU-Split	95	90	90-0-90
34	Männlich	FCU-Split	100	150	90-0-90
63	Weiblich	Kocher + FCU-Split	100	125	90-0-90
51	Weiblich	Kocher + FCU-Split	n. verf	n. verf	n. verf
63	Weiblich	Kocher + FCU-Split	n. verf	n. verf	n. verf
22	Männlich	Kocher + FCU-Split	100	140	80-0-80
52	Weiblich	Kocher + FCU-Split	95	70	70-0-60
76	Weiblich	Kocher + FCU-Split	100	140	80-0-80
65	Weiblich	Kocher + FCU-Split	100	125	80-0-80
61	Weiblich	FCU-Split	95	90	90-0-90
